# Handgrip Strength in Health Applications: A Review of the Measurement Methodologies and Influencing Factors

**DOI:** 10.3390/s24165100

**Published:** 2024-08-06

**Authors:** Antonino Quattrocchi, Giada Garufi, Giovanni Gugliandolo, Cristiano De Marchis, Domenicantonio Collufio, Salvatore Massimiliano Cardali, Nicola Donato

**Affiliations:** 1 Department of Engineering, University of Messina, 98166 Messina, Italy; giovanni.gugliandolo@unime.it (G.G.); nicola.donato@unime.it (N.D.); 2 Department of Neurosurgery, Azienda Ospedaliera Papardo, University of Messina, 98158 Messina, Italy; giadagarufi@hotmail.it (G.G.); domenicantonio.collufio@unime.it (D.C.); scardali@unime.it (S.M.C.); 3 Division of Neurosurgery, BIOMORF Department, University of Messina, 98124 Messina, Italy

**Keywords:** handgrip strength, measurement issues, instruments and devices, methodological aspects, measurement protocols, parameters of interest, endogenous and exogenous factors, health application measurements

## Abstract

This narrative review provides a comprehensive analysis of the several methods and technologies employed to measure handgrip strength (HGS), a significant indicator of neuromuscular strength and overall health. The document evaluates a range of devices, from traditional dynamometers to innovative sensor-based systems, and assesses their effectiveness and application in different demographic groups. Special attention is given to the methodological aspects of HGS estimation, including the influence of device design and measurement protocols. Endogenous factors such as hand dominance and size, body mass, age and gender, as well as exogenous factors including circadian influences and psychological factors, are examined. The review identifies significant variations in the implementation of HGS measurements and interpretation of the resultant data, emphasizing the need for careful consideration of these factors when using HGS as a diagnostic or research tool. It highlights the necessity of standardizing measurement protocols to establish universal guidelines that enhance the comparability and consistency of HGS assessments across various settings and populations.

## 1. Introduction

Hands are an essential tool for humans and have played a significant role in our evolution. They can perform movements related to grasping and moving objects, and, specifically, two types of handgrips can be distinguished: power and precision [1,2]. Power, or palmar, handgrip consists of flexing the fingers to allow us to grasp heavy objects and for their large displacements, as well as to hold on to something and maintain our body position. Precision grip, on the other hand, involves the closing the fingers, especially the thumb and forefinger, in order to manipulate small objects with a high level of detail.

Handgrip strength (HGS) is commonly used to estimate power handgrip. It is considered to be a general indicator of muscle strength and power and is useful in assessing the overall health status when combined with other biological parameters [3,4,5,6,7,8]. Furthermore, it is commonly assessed by measuring one main parameter, the maximum isometric force (F_max_) or maximum voluntary contraction (MVC), using a dynamometer [9,10,11,12].

A sufficient, if not high, level of HGS is necessary for ordinary functions of daily life, but it is also essential for many sports and work activities. HGS is a reliable index for assessing the characteristics of athletes and workers in a wide range of activities, including climbing [13], martial arts [14,15], volleyball [16,17], football [18], swimming [19], basketball [20], sports and common driving [21,22,23] and manual activities in agriculture [24], allowing for an instrumental comparison between different individuals. In the medical field, HGS estimation is versatile; it may be used to ensure the proper conduct and effective results of rehabilitation processes [25,26,27], to guarantee the effectiveness of treatments for musculoskeletal disorders of the hand [28,29], to assess clinical conditions in musculoskeletal and neurological pathologies [30,31,32,33], especially of the upper limbs [34], to allow optimization of hand–arm prostheses [35] and also to determine the general health status of individuals with other clinical conditions [36,37]. HGS also serves as a consistent screening technique for specific diseases: Lopes et al. [38] conducted a comprehensive analysis of 413 patients, 165 females and 248 males, with 299 aged <60 years and 114 ≥60 years who had been on dialysis for less than 6 months. This study provides further evidence to support HGS as a valid nutritional screening tool and asserts that assessment of HGS within the first few months of hemodialysis initiation may identify patients at a higher risk of death. Eckman et al. [6] highlighted that, in their sample of 90 diabetics and 46 non-diabetics aged from 20 to 88 years, diabetics had lower HGS. As a result, a simple binary logistic model using HGS, age, blood pressure and body mass index (BMI) was proposed to confidently predict a patient’s likelihood of having diabetes.

In addition, HGS, when combined with other biomedical measurements, provides important information for understanding neuromuscular fatigue processes [39,40,41,42] and for estimating human organ and system performance [43,44,45,46,47,48,49,50,51]. Di Rienzo et al. [52] investigated the effects of psychophysical stress on cardiological parameters associated with wrist movement. The study involved a series of physical exercises including handgrip contractions at 30% of MVC for 3 min. Korte et al. [9] evaluated the hemodynamic function of the pathological left ventricle in patients at rest and during exercise. They selected different types of handgrip exercises with a duration of 2–4 min. Phillips et al. [53] studied the electromyographic signals after applying a fatigue model for isometric squats and handgrip at 30% of MVC for 2 min. In most of these studies, the exercise models were varied based on the MVC value, e.g., 15–30% [54], 45% [55] or 50% [56,57], and therefore no common standard exists.

Among the specific applications, HGS is used to indirectly estimate anthropometric parameters, such as the gait cycle [58], to develop training programs, often for elderly subjects with critical pathologies [59], to evaluate electromyography of the arm muscles [21,60] and to design assistive devices for diseases involving the spinal cord [22] and hand tremor [61]. Finally, Le et al. [62] found a strong to moderate relationship between HGS and chance anticipation timing in young adults. This study included 23 females and 1 male between the ages of 17 and 27.

Although there is a large amount of literature focusing on HGS, few studies have examined aspects of its measurement and standardization of related methods. In addition, most of the research lacks homogeneous information and tends to focus on specific cases that are difficult to generalize.

The aim of this work is to report and critically analyze the current state of the technical engineering literature on HGS and its measurement characteristics, including the devices and equipment used, the measurement methods, the extracted metrics and the possible factors affecting the measurement. This paper includes classical and innovative devices, endogenous and exogenous variables and, finally, the techno-practical methods that can influence such measurements. This information can be useful for researchers and medical professionals who work in clinical settings, as well as for coaches and athletes who apply these considerations in sports. It is important for all scientists to strive for an objective, quantitative, simple and standardized evaluation of HGS measurements.

The documents discussed in this narrative review were selected from the main scientific databases (Scopus, Web of Science and PubMed) in January 2024. Initially, articles were included if they were published in English, with engineering as the subject and in journals between 2000 and 2023. Then, the search terms “handgrip strength”, “handgrip measurement”, “handgrip instrument”, “handgrip device”, “handgrip prehension force”, “handgrip force” and “hand grasp force” in the keywords, title or abstract were considered. These were chosen to emphasize the measurement character of our paper by incorporating the parameters of interest (strength and force), the topic of interest (measurement) and the measurement equipment (instruments and devices). Finally, studies were included in the review if they met the following inclusion criteria:

1. The primary focus of the study was methodological, encompassing the development of an HGS device, the examination of measurement procedures and the investigation of methodological aspects related to data processing.
2. The proposed device, measurement procedure or processing methodology was subjected to testing in real experimental conditions.

The review is organized as follows: Section 2 is focused on the main measurement issues raised in the literature and on the common and innovative equipment employed for HGS tests. Section 3 presents methodological aspects related to HGS estimation. Section 4 and Section 5 deal in depth with endogenous and exogenous factors affecting HGS measurement. Finally, Section 6 summarizes the main findings of the review, discusses the relevant limitations of current HGS measurement practices and concludes by emphasizing the need for debate and critical attention toward measurement aspects related to HGS.

## 2. Issues and Measurement Techniques for Handgrip Strength

Several studies have attempted to establish standards for determining the most representative values for HGS. However, to date, a large number of protocols and related data have been reported in the literature. These refer to a wide collection of experimental methods, measurement instruments and quantitative parameters for HGS estimation. Instrument design and sensing have a significant impact on the measurement, influencing data acquisition and often limiting the registration of the parameters of interest. Further variability is introduced by all the procedures of the adopted protocol, such as the subject’s position and limb placement during the test (e.g., global posture, arm flexion, instrument grasp), the entire acquisition procedure (e.g., duration and amplitude of the exerted force) and the choice of parameters of interest (e.g., maximum force). In addition, intrinsic and extrinsic aspects of the subject should be taken into account. Gender and age are the primary factors to be considered, but also the dominance of the tested hand and the general biological characteristics of human systems (e.g., hand size and body mass) play a relevant role. Environmental influences (e.g., time of day of the measurement, work activity of the subject, smoking or not and psychological effects caused by external stimuli) are further discussed.

**Figure 1:**
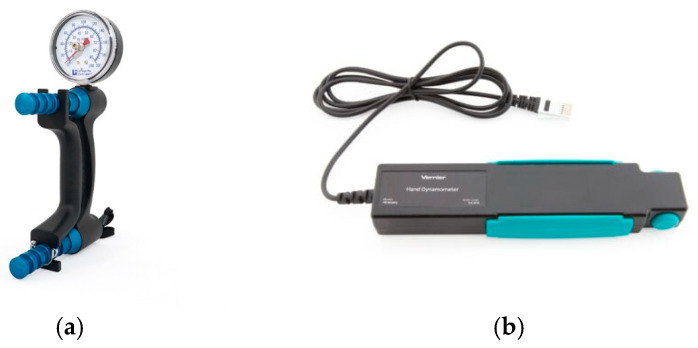
(**a**) Hydraulic dynamometer [65] and (**b**) strain-gauge-based electronic dynamometer [66].

Currently, HGS is typically measured using two types of dynamometers (Figure 1), which differ in their sensing element. The first type [63], known as a hydraulic dynamometer (although this term is often misused), uses a load cell. The latter is embedded in a device consisting of a sliding handle, which is compressed against a fixed handle during gripping. Because of this design, the size of the load cell needs to be relatively small. The measuring principle is based on the conversion of the pressure exerted by the hand into a gripping force. The second type [64], defined as an electronic dynamometer, uses a force-sensitive resistor (FSR). A precise interaction between the instrument, the fingers and a specific area of the palm is required to obtain representative results. This technology is typically found in ergonomic devices such as gloves or custom grips. As a measurement principle, HGS is calculated by monitoring the impedance change in an electronic circuit attached to the grip holder caused by the grip force.

In addition, these devices are capable of measuring isometric forces, i.e., with muscle contraction but without macroscopic muscle elongation, and isokinetic forces, i.e., at a constant and controlled angular velocity within the established range of motion. Traditionally, dynamometers have been used primarily to assess isometric HGS [67,68,69,70,71].

### 2.1. Traditional Measurement Devices

Among the many devices on the market today (Table 1), the load-cell-based Jamar dynamometer (Patterson Medical Ltd., Sutton-in-Ashfield, UK) is widely used and considered in many applications [72,73,74,75]. Nikodelis et al. [76] compared two dynamometers, a classic Jamar Plus and a more innovative K-Force Grip Kinvent with tubular ergonomics. These devices have different design characteristics, such as size and mass (60 mm × 140 mm × 240 mm and 490 g for Jamar, 40 mm × 45 mm × 120 mm and 150 g for K-Force Grip) and especially the handle position (adjustable for Jamar and fixed for K-Force Grip) for the subject. A sample of 113 people, divided into healthy, shoulder pain, male, female, young and elderly, gave different results, which the authors attributed to the different handgrips.

### 2.2. Innovative Measurement Devices

In the context of innovative devices, several measuring instruments based on advanced techniques and/or suitabitity for unconventional applications are present. Mohaini et al. [94] proposed an instrumented bicycle handlebar for children with autism spectrum disorder. This device was designed in the Internet of Things (IoT) field to perform measurements on patients who have difficulty following a set of procedures, and to monitor the results of the same measurements via Wi-Fi on a cloud system. The device comprises a load cell for measuring HGS, a heart rate sensor and an ESP32 board with dedicated electronics to condition the results, which are placed in a 3D-printed dumbbell. Kaidi et al. [95] developed an IoT prototype to remotely monitor a post-stroke patient’s rehabilitation process using Arduino Nano and FSR. Specifically, it is a cylinder with a diameter of 30 mm and length of 150 cm, comprising six measurement zones that provide an accuracy of ±1% for forces up to 250 N. Whang et al. [68] presented a new HGS estimation method oriented toward the multi-wrist angle based on the development of a flexible deformation sensor. The flexible deformation sensor comprises a foaming sponge, a Hall sensor, an LED and photoresistors. When external pressure is applied to the foaming sponge, its density and light intensity change, which are detected by a light-sensitive resistor. The system was calibrated using an HX711 force sensor. Jahan et al. [96] developed a handgrip using an optical fiber Bragg grating. This device converts the grip force exerted at a surface into strain variation on the vertical bars, which is sensed by bonded FBG. Its modeling was performed using Ansys Multiphysics, while its calibration procedure was carried out using a micro universal testing machine. Finally, Van Drongelen et al. [97] developed an instrumented handbike system to quantify the forces applied to the handgrip during handbiking. A six-degree-of-freedom force sensor, based on strain gauges, was integrated into the handgrip of an attachment unit handbike. The instrumented handbike system demonstrated excellent linearity in both static and dynamic conditions. However, precision decreased with higher loads during the dynamic condition. Finally, the percentage error values ranged from 0.3% to 5.1%.

### 2.3. Alternative Methods for Force Measurement

In addition to the traditional HGS measurements, alternative methods and consequent parameters have been identified. Rahman et al. [98] and Mohd Fa’iz et al. [99] conducted evaluations and mapping of the pressure exerted by hands, fingers and palms on the steering wheel during specific vehicle driving maneuvers. A commercial piezoresistive matrix system was employed for the collection of data, which was then compared on two types of steering wheels. Although of certain interest, the evaluation of grip pressure does not appear to be an adequate method for estimating HGS. Moreover, the instrumentation employed is more expensive and complex than traditional dynamometers. Yokoyama et al. [100] demonstrated a correlation between HGS and surface electromyography data. They developed an elastic glove with dry electrodes that are capable of measuring signals from the back of the hand. The acquired data were then compared with that obtained using a classical dynamometer and processed using artificial neural network regression models. The results demonstrated a good correlation (ranging from 0.770 to 0.840, depending on the specific method employed) between the predicted forces and observed forces, which were normalized by the MVC for each subject. Due to the limited correlation, the authors concluded that the investigated device should be proposed only for nonclinical measurements. In their study, Santos Borges et al. [101] analyzed two parameters, explosive force and contractile impulse, which are derived from the slopes of moment/time curves during the first 200 ms of contraction. Fuss et al. [13] developed an instrument based on four tri-axial piezoelectric force transducers to measure the individual vertical and normal finger forces during a climbing activity. Pylatiuk et al. [102] proposed a similar study, employing a modified standard conductive polymer pressure sensor for force-pressure transduction. In all cases, the provided estimations did not cover handgrip force and required specific instruments that are not comparable with classical dynamometers. Paul et al. [103] designed and validated a device employing fiber Bragg gratings (FBGs) to measure handgrip force. Specifically, five FBGs, realized at different center wavelengths on a single photosensitive fiber, were utilized to obtain the responses from the individual fingers. This device exhibits a superior sensitivity in comparison to FSR ones, and, due to the incorporation of FBGs, it is not susceptible to electromagnetic and temperature-related influences. While it is of interest to highlight the different contributions of the fingers, it should be noted that this measurement does not provide a suitable HGS.

## 3. Methodological Aspects

In the scientific literature, HGS measurements are frequently reported in accordance with the guidelines established by associations with expertise in sports and medical issues. Nevertheless, researchers in this field frequently present a number of procedural variations.

### 3.1. Measurement Protocols

In 1981, the American Society of Hand Therapists (ASHT) [104] recommended the use of a dynamometer with a size corresponding to that of the hand holding it. To measure HGS, subjects should remain seated in a chair without armrests with the spine erect, the knees flexed at 90°, the shoulders positioned in adduction and neutral rotation, the elbows flexed at 90°, the forearms in half pronation and the wrists in a neutral position, with the possibility of moving them up to 30° degrees of extension. The arm under testing should be held in a suspended position with the hand placed on the dynamometer, supported by the evaluator. The measurement of HGS was expressed in kilograms (kg). This procedure is well established in the literature and is applied in numerous works, including those by [23,41,105,106], but recently, some differences have emerged. Hsu et al. [40] introduced variations to the procedure, including the use of only the dominant hand and the placement of the hand on a supporting table, with the dynamometer held freely. Conversely, Majstorović et al. [17] elected to compute HGS for both hands, the right and left, and to report the measured data in newtons (N). Florianovicz et al. [107] proposed additional modifications to the previous procedure. These included adapting the dynamometer to the subject’s hand size, ensuring that the most distal finger joints fit the handle; prohibiting hand movements during the measurements, as these could alter the results; and instructing the subjects to focus mentally on the task required for HGS measurement.

In other protocols, the patient is required to sit with the arm extended laterally [108] or to stand with the arm extended along the side [109], to grasp the dynamometer, to apply the grip as quickly as possible and then to repeat the procedure with a rest period in between. In a study by Gil et al. [10], the reproducibility of two dynamometers in the handrail format for measuring HGS and traction force in young and older adults was estimated. To assess HGS, each participant was positioned with the legs laterally displaced (shoulder width) and the hand performing the grip in the center of the dynamometers, with the elbow flexed at 90°.

A subject’s choice of the number of repetitions to be carried out is of particular interest. In the literature, HGS is typically evaluated by adopting an average value or the best performance. In contrast, Legg et al. [72] and Tyagi et al. [12] opted to average three measurements, although they employed different protocols. Florianovicz et al. [107], Zhu et al. [109] and Patel et al. [67], meanwhile, selected the best measurement from three attempts. In contrast, Schibye et al. [110] decided to repeat the test three times, with the third repetition excluded if it exceeded 5% of the highest value obtained in the previous two repetitions. Finally, Schmidt et al. [111] and Öcal Kaplana [20] conducted the test on the non-dominant hand only, while Nowak et al. [18] performed two tests for the right and left hands.

Another factor to be considered is the total duration of the measurement. Typically, the grip is maintained for a few seconds, e.g., 3 s [64], 4 s [28] or 5 s [72], while in the case of multiple measurements, a resting phase is employed. The latter can vary in duration from a few tens of seconds, e.g., 10 s [111], 15 s [36] or 30 s [72], to a few minutes, e.g., 1 min [37], 2 min [10] or 3 min [112].

### 3.2. Parameters of Interest and Force Signal Analysis

The parameters of interest in the estimation of HGS are numerous and are largely dependent on the applied experimental procedure. As previously stated, the measurement of F_max_ or MVC in kilograms or newtons is the most commonly employed approach. Indeed, many studies consider the peak force (100% of F_max_) reached within a defined time interval. In other instances, however, they refer to a specific fraction of F_max_, typically between 20% and 90% [72,109,111,113]. The regular maximum, mean and minimum values of F_max_ are not standardized. As reported by Wang [36], the Asian Working Group for Sarcopenia (AWGS) [114] considers an HGS <26 kg for males and <18 kg for females to be indicative of low muscle strength. In the case of sarcopenia, these values even decrease to <18 kg for males and <16 kg for females. The same criteria have also been employed by the Japan Society of Hepatology [95]. In neurological diseases such as chronic stroke and multiple sclerosis, the values are further reduced, from 7.8 kg to 16.5 kg and from 9.1 to 16.2 kg, respectively [115]. In general, F_max_ is a relatively straightforward and rapid indicator to identify. Nevertheless, in clinical, sports and even scientific practice, additional indicators found between the beginning and end of the grip are of interest.

A dynamometer for measuring HGS is well described by the model of a first-order instrument with a one-step response. In fact, when a constant grip force is applied as the input, the force measured as the output is characterized by a typical increase in a nonlinear trend, i.e., a transient response, until a plateau is reached, i.e., a steady-state response [11]. When the subject maintains the grip, the force reaches its maximum value, F_max_, and then physiologically decreases slowly to the final force, F_final_. This behavior is sufficiently symmetrical, and approximately linear, but exhibits a slight imbalance towards higher values in the initial section. In the absence of any external constraints, the force instantaneously reaches zero [72].

**Figure 2:**
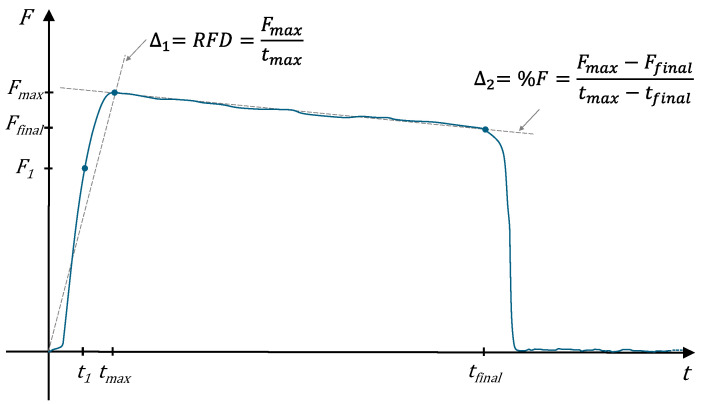
Typical trends of the individual’s applied force during an HGS test. The dashed lines represent the slopes relative to Δ_1_ and Δ_2_.

In 1980, Myers et al. [116] proposed F_max_, F_final_, the rate of force development (RFD) between 0 N and F_max_, the rate of force loss (%F) between F_max_ and F_final_, and the work (L) as the area under the curve. Recently, Urbano et al. [11] expanded upon the previous considerations by introducing additional parameters. These include the 67% of F_max_ (F_1_), the times corresponding to F_1_, F_max_ and F_final_ (respectively, t_1_, t_max_ and t_final_), the rate of force development until F_max_ given by Δ_1_ = F_max_/t_max_, which is equivalent to the previous RFD, and the subsequent rate of decay until the end of the test, defined as Δ_2_ = (F_max_ − F_final_) / (t_max_ − t_final_), which is equivalent to the previous %F. Figure 2 presents a schematic representation of the parameters under consideration.

**Figure 3:**
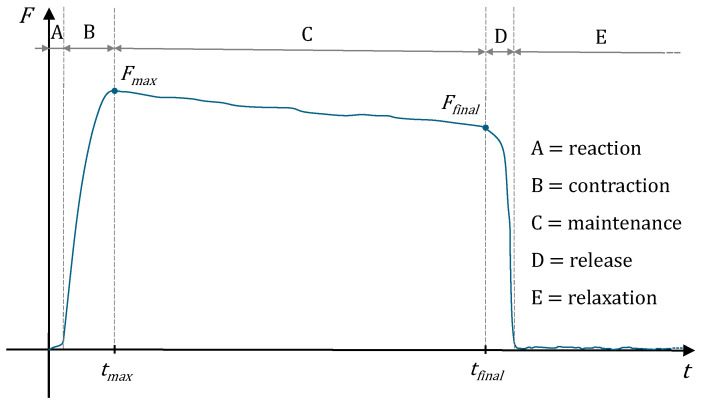
Phases of an HGS test compared to the force trends.

As reported by Andria et al. [117], the acquired force signal can be divided into five main phases: reaction, contraction, maintenance, release and relaxation (Figure 3). The duration of each phase and the shape of the recorded signal are contingent upon a multitude of variables. Some of these factors, such as reactive and auditory ability, anxiety and mood, are not within the subject’s control. In contrast, factors such as high tremor, reaction speed and maximum strength level depend on the subject’s state of health.

### 3.3. Device Grip

The distinctive design characteristics of the measuring devices, particularly the handles through which the force is applied, may influence the subject’s grip or vice versa. The literature contains a great deal of discussion regarding the adjustment of the size of the handle used in the grip dynamometer. The distance between the fingertips and the palm can significantly affect the grip and the force exerted. For this reason, ASHT and a significant number of researchers have emphasized the importance of adapting the appropriate equipment to different hand sizes in order to obtain an objective estimation of the parameters of interest [5,39,103,118,119]. Another aspect to consider should be the correct interaction between the hand and the measuring instrument. Nataraj et al. [120] have demonstrated that materials that exhibit various different characteristics, such as compliant surfaces, guarantee a different grasp force, which can be optimized through visual feedback.

### 3.4. Arm and Elbow Positions

The arm and elbow positions serve as accommodating factors for the intrinsic regulation of the intensity of the force exerted by the subject. In a study by Restrepo-Correa et al. [121], three arm positions were compared using an adjustable-angle support. The statistical analysis of the measurements of 32 participants with a mean age of (23.1 ± 3.6) years revealed that the use of an arm support affects HSG estimation. However, the angular variation of the support, and thus of the arm itself, did not produce a significant incidence. Marković et al. [122] evaluated the inter-reliability, concurrent validity and interchangeability of an SMS HG and a Jamar dynamometer for HGS measurements, operated in two elbow joint positions: 90° flexion and 180° extension. The study, conducted on 61 participants, demonstrated that the measurements obtained in the same elbow position were comparable between the two instruments. However, when the position was varied, the maximum difference was 8.84%. Aziz et al. [123] examined the impacts of three distinct elbow flexion angles (0°, 90° and 180°) on 200 female students between the ages of 18 and 25 years. The results indicated that the greatest force was produced when the handgrip was performed with 0° elbow flexion, specifically (27.22 ± 3.79) kg with the dominant hand and (25.48 ± 3.42) kg with the non-dominant hand. (In the whole paper, the first value within parentheses represents the mean, while the second one is the standard deviation.) Finally, Khader et al. [37] analyzed the maximum HGS of 100 participants in seven different anatomical arm positions. The recorded values were found to be significantly influenced by the angle of the arm, as well as by anthropometric aspects, weight and gender.

### 3.5. Calibration

The majority of dynamometers currently available on the market lack the capacity for user-performed calibration. Consequently, it is recommended that the device be sent to the manufacturer or a calibration center for servicing after a period of one year. In the event of frequent use, calibration should be conducted at an shorter interval, and the need for calibration should be evaluated by the operator [119].

## 4. Endogenous Factors Influencing Handgrip Strength Value

A number of factors related to the individual subject’s physiological characteristics must be taken into account when assessing HGS. These characteristics are of pivotal importance in determining the most appropriate measurement method and should be evaluated in a rigorous manner to define the corresponding procedural considerations.

### 4.1. Hand Dominance

In general, the natural preference of a limb, and consequently of a hand, tends to result in greater strength and dexterity. Indeed, Haddix et al. [113] demonstrated that the use of the right or left hand is determined by symmetrical muscles and separate areas of the brain, though they had a limited sample size of 14 participants. Handgrip force [124] is not solely determined by the musculature of the same hand. It is also significantly influenced by that of the correspondent arm. The muscles of the latter can exert a distinct effect when used more frequently on either the right or left side. Consequently, this ensures markedly disparate behaviors and outcomes.

In 1954, Bechtol et al. [125] established the “10% rule”, which states that the dominant hand has 10% greater grip strength than the non-dominant hand. A more recent study by Aziz et al. [123] found that in a sample of 200 women aged 18 to 25 years, the grip strength of the dominant hand was greater than that of the non-dominant hand at different elbow flexion angles. The maximum observed difference was 7.03%. Further confirmation was provided by Kharb et al. [24], who analyzed a sample of 173 male farmers and found that HGS in the right hand was 3.6% higher than the other hand. Öcal Kaplan [20] made a significant contribution to the field by investigating 101 active athletes, 49 females and 52 males, with an average age of (20.00 ± 1.42) years in females and (21.00 ± 1.99) years in males. The results of the analysis indicated that the dominant and non-dominant HGSs of athletes in certain disciplines and genders exhibited significant differences. In particular, the mean HGS for male basketball players was 48.25 ± 5.42 kg for the dominant hand and 53.84 ± 6.39 kg for the non-dominant hand. Instead, female basketball players exhibited a mean HGS of 32.00 ± 4.04 kg for the dominant hand and 28.81 ± 3.18 kg for the non-dominant hand. In contrast, Pawlik et al. [16] observed that in young female volleyball players, the difference between right-handed and left-handed grips was essentially not relevant. Specifically, in 12 girls aged 12–13 years, the difference between right-handed and left-handed grips was (27.3 ± 3.1) kg vs. (27.6 ± 3.4) kg, respectively.

### 4.2. Hand Size

The dimensions of the human hand can have practical and technical implications in a variety of fields. For instance, hand size and resulting body proportions are employed to assess HGS and, in turn, to determine the design specifications of agricultural equipment [126]. Furthermore, Ramos et al. [127] demonstrated that anthropometric variables such as hand width, hand aperture and hand length also have a particular influence on adolescents. The findings of these studies indicate an increase in the capacity to exert HGS during adolescence, with a notable disparity between the sexes emerging at 13 years of age. Specifically, boys exhibited higher HGS values than girls.

### 4.3. Body Mass

It is common to examine the influence of a subject’s body mass, specifically BMI, on the estimation of HGS. In a study by Krzysztoszek et al. [128], the relationship between physical activity and the fitness level was evaluated in 82 obese participants, 48 women and 34 men, over 40 years of age with coexisting hypertension. The results led the authors to conclude that an increase in physical activity, and thus a reduction in BMI, corresponds to a subsequent improvement in HGS. In a study of 2538 healthy participants, comprising 1215 women and 1323 men aged 40 to 69, Jeon et al. [80] examined the correlation between HGS and metabolic syndrome. The investigation was conducted over a 16-year period. The findings indicated that a low HGS, which is correlated with a high BMI, is associated with an elevated risk of developing a metabolic disorder in the future.

In 1995, Vanderburgh et al. [129] proposed the use of an allometric scale to derive the correct values of force (F_corr_) as a function of the subject’s body mass (MC), thus allowing for a suitable comparison. The suggested equation is

F_corr_ = F_max_ × MC^b^ (1)

where “b” is the allometric adjustment exponent. In their study, developed on 279 American and English students, these researchers recommended a “b” of 0.51 for both genders.

Godziuk et al. [130] estimated the prevalence of sarcopenic obesity in adults with end-stage knee osteoarthritis. The study included 151 participants, 59% of whom were female, with a mean age of (65.1 ± 7.9) years and a mean BMI of (37.1 ± 5.5) kg/m^2^. The results indicated that the estimation of HGS in conjunction with additional parameters, such as weight and BMI, represents a valuable diagnostic tool. In a separate study, Mehta et al. [92] investigated the influence of obesity and age on handgrip endurance across a range of relative workloads. A total of 45 non-obese and obese younger and older women performed fatiguing handgrip exercises at 20%, 40%, 60% and 80% of MVC. The younger obese group exhibited approximately 7% greater strength, 32% shorter endurance times and an approximately 34% faster rate of strength loss, accompanied by a heightened perception of effort, than the younger non-obese group. However, these differences were not observed in the older age group.

### 4.4. Gender and Age

In general, when other factors are held constant, males tend to have a higher HGS than females ((19.12 ± 10.65) kg vs. (10.44 ± 6.24) kg, respectively [126]), and these findings are observed in each age group [131]. Patel et al. [67] conducted a study involving 200 agricultural workers, 130 males and 70 females, with an age range from 17 to 62 years. The results of Student’s *t*-tests indicated significant differences in HGS between male and female workers. The mean HGS for the right hand was (30.11 ± 7.06) kg for males and (19.75 ± 5.38) kg for females, while for the left hand it was (26.59 ± 6.84) kg for males and (15.96 ± 5.74) kg for females. In a study of 70 female participants aged 18 to 25 years, Isik et al. [41] found that the menstrual cycle has no influence on HGS, but significantly affects attention and manual dexterity for tool use. In contrast, Wen et al. [132] identified the possibility of early diagnosis and assessment of the status for diseases such as sarcopenia by estimating HGS in postmenopausal women.

It is also evident that HGS is directly related to age. Indeed, this parameter exhibits a gradual increase until a defined age, after which it declines significantly. In a study by Majstorovic et al. [17], a model was developed to determine the age-related contractile characteristics of multidimensional muscles in volleyball players. The investigation included 483 participants, 112 males and 371 females, divided into four age groups (under 15, under 17, under 19 and under 21). Significant differences were observed between the genders at the same age, with males exhibiting (572.3 ± 82.7) N and females (347.4 ± 55.2) N. These values were obtained from individuals under 21. Additionally, significant differences were observed within the same gender, with males demonstrating a 78% increase and females a 27% increase from under 15 to under 21. Kharb et al. [24] observed that for a group of 82 male farmers between the ages of 20 and 29 years, the dominant HGS was (47.52 ± 6.69) kg. However, with increasing age, this value decreased to (40.75 ± 5.92) kg for a group of 29 male farmers between the ages of 40 and 50 years. Indeed, older participants [133], over the age of 65 years, can exhibit remarkably low HGS values, as low as (15.00 ± 6.35) kg, which can be improved through periodic training. Schubert et al. [134] investigated the behavior of eight older individuals (four males and four females, aged 68.1 ± 5.2 years), subjected to accelerations and decelerations on a bus. The results demonstrated that the capacity to use bus transportation is contingent on psycho-physical prerequisites that are strongly correlated with the age. These abilities exerted a significant influence on handgrip prehension force in module and duration of the grip. Wu et al. [63] analyzed a sample of 20 participants, who were divided into four groups based on age: young ((22.00 ± 1.26) years), older ((51.70 ± 6.24) years), adult males and adult females. It was observed that with age, the strength signal, as well as the electromyographic signals related to certain arm muscles, tend to lose complexity and undergo a significant reduction in general. In particular, the maximum isometric HGS ranged from approximately 180 N in the older subjects to approximately 110 N in the younger ones at 75% of the MVC. Roman-Liu et al. [7] investigated the relationship between HGS and force moments in the joints of the upper and lower limbs in relation to age and gender. The study demonstrated that, in general, the greatest age-related differences in muscle strength occurred in the lower limb muscles. However, for men, age primarily affects upper limb strength, while for women, it affects lower limb strength. Indeed, a woman’s HGS is more susceptible to age-related decline than a man’s [118].

## 5. Exogenous Factors Influencing Handgrip Strength

The evaluation of HGS may also be influenced by external sources that are often unconscious and difficult to predict. Such factors may include environmental conditions, lifestyle choices and psychological concerns.

### 5.1. Moments of the Day

Throughout the day, the physiological characteristics of each individual undergo changes that depend not only on intrinsic aspects but also on external stimuli. In 1984, McGarvey et al. [135] observed differences in F_max_ ranging from 5% to 7% between different times of the same day in 40 normal subjects with ages from 40 to 70 years. In a recent study, Matveev et al. [8] identified potential changes in the cardiac autonomic balance between the morning (8–9 a.m.) and afternoon (2–3 p.m.) in 22 healthy participants, comprising 11 men and 11 women with a mean age of 47.3 years (ranging from 21 to 76 years). The participants had no history of cardiovascular or neurological diseases, organ insufficiency or diabetes. The data indicated slight variations in specific strength parameters.

### 5.2. Job/Occupation

The job/occupation is a key determinant of the subject’s physical and mental functions, which in turn influence the grip strength exerted. Jain et al. [136] observed 182 manual workers, comprising both men and women, grouped according to three levels of experience. Their findings indicated that upper limb muscle activity and the use of tools for specific manual tasks have a significant impact on HGS. Furthermore, they observed that professionals who adopt a neutral position tend to exert higher forces. Saremi et al. [118] investigated the relationships between dentists’ hand function, specifically HGS, anthropometric hand size and their occupation. The study included 720 dentists, 330 males and 390 females. The results demonstrated that hand function was strongly related to specialization and clinical experience. In a separate study, Tokarski et al. [137] analyzed a sample of 52 women divided into three age groups: 20–25, 45–50 and 55–65 years. They observed that the forces exerted in the workplace by older workers are approximately 20% lower than those exerted by workers aged 20–24 years. However, when the tasks engage small muscles of the forearms and hands, such as assembly or manipulation work, older workers have similar force capabilities to young workers. Hsiao et al. [112] examined the muscle strength and performance fatigability of the forearms in eight male orthopedic surgeons aged between 28 and 40 years, when performing bone screw fixations. Pre- and post-fatigue F_max_ and corresponding electromyography responses were measured. The results demonstrated that, following the insertion of eight bone screws, F_max_ was approximately 29%. The driving forces exhibited a decreasing trend, while the insertion time had a parabolic increase with the number of screw insertions. Chandra et al. [138] measured the HGSs of 45 subjects, who were equally divided into three groups: safety inspectors, industrial workers and video display terminal operators. The results of the evaluation demonstrated a significantly lower HGS value in industrial workers (42.6 ± 4.1) kg and video display terminal operators (23.6 ± 5.9) kg compared to safety inspectors (48.4 ± 5.5) kg. The authors posited that this may be attributed to the stressors inherent to the job, particularly those associated with repetitive tasks and suboptimal work environments.

### 5.3. Smoking

It is important to consider the impact of smoking on overall health, with particular attention to its effect on muscle strength. As indicated by Bani et al. [106], smoking has been demonstrated to influence HGS in relation to the age of the individual and the duration of the smoking habit. The study revealed that subjects who had been smoking for over 10 years exhibited a lower HGS than those who had never smoked or who had quit smoking for over 10 years. Furthermore, individuals who had smoked for over 50 years demonstrated an HGS between 8 kg and 10 kg, regardless of their age category.

### 5.4. Psychological Sphere

The final aspect to be highlighted pertains to the psychological domain of the individual. Here, the effort can depend on aspects related to the motivation, the comprehension of the test procedure and the discomfort caused by the use of a specific dynamometer. Given the difficulty in controlling these relative variables, it is recommended that the investigated person be familiar with the dynamometer in order to understand the test phases. For this reason, the use of verbal encouragement [105,110] or audio-visual feedback [36,109,113] can significantly influence the results of measurements of HGS. For instance, Garcia-Hernandez et al. [28] provided extensive instructions to patients regarding the measurement procedure, provided postural feedback in front of a mirror and recorded the achieved HGS value. In contrast, Andria et al. [117] employed an acoustic start and end signal for the test. This approach is more comfortable, particularly for patients with Parkinson’s disease, who were the subjects of this study.

## 6. Discussion and Conclusions

This review provides a detailed analysis of the methods and technologies employed to measure HGS, which is recognized not only as an indicator of neuromuscular strength but also as a critical health metric related to functional capacity and disease prognosis. The findings of this review are as follows:

(1) Issues and measurement techniques—This paper covers several devices, from traditional to novel, highlighting the importance of methodological rigor and technological advancement in the accurate assessment of HGS. Although traditional dynamometers lack a standard geometric shape, as highlighted in Section 2, they all have a fixed handle, a sliding handle, a load cell and a display or indicator. The working modes are similar: the patient rests their palm against the fixed handle, while their fingers wrap around the sliding handle and exert the grip that is measured by the load cell and shown on the display or indicator. From a purely geometric perspective, the discrepancies between devices from multiple manufacturers are mainly attributable to the widths of the individual handles, the distance between two handles, the shaping for the palm on the fixed handle and for the fingers on the sliding handle, and the overall volume. However, the weight of the handgrip device can also vary depending on the materials used and the construction stiffness adopted. These parameters are of significant importance for the suitable handling of the device, but more prominently, they can largely influence the correct execution of the test. Indeed, in the case of subjects with either small or large hand sizes, not all handgrip devices are adaptable and only a limited number of fit levels can be selected. Moreover, for patients who have functional limitations or impairments of one or more fingers or even parts of the hand, it is necessary to consider specific customized devices. Finally (see Table 1), from a purely measurement point of view, there is a limited variability within the measurement range (±10 kg, 80 kg to 100 kg except for MAP, mod. 130K1, for which the maximum measurable value is 130 kg). However, the differences in accuracy (up to 400%, from ±0.5 kg to ±2.0 kg) and especially in resolution (up to 10 times, from 0.1 kg to 1 kg) become more significant. These metrological properties should be carefully considered since they are responsible for the adequacy of the measurement and are difficult to distinguish by an inexperienced operator and the patient. Finally, each manufacturer adopts its own design, which can significantly differ from others, leading to variations in measurement results even under similar test conditions. This diversity makes it difficult to establish universal measurement protocols and compromises the consistency of data collection and analysis across studies and applications.
(2) Methodological aspects—This study has emphasized some limitations of current HGS measurement practices. The choice of a representative parameter for the measurement of HGS is therefore a topic of considerable discussion in the literature, and a common and objective strategy has not yet been identified. Although the maximal isometric force F_max_ (see Figure 2) is relatively straightforward to consider, this value tends to exhibit a rapid decay and frequently differs significantly from its final value F_final_. Similarly, the evaluation of a fraction of F_max_ does not ensure a broadly agreeable outcome. It could be really interesting to estimate a generalized parameter that takes into account F_max_, i.e., an impulsive force, and the duration of the measurement, i.e., a muscular endurance. For these reasons, although it is rarely considered in the field of sports and medicine, the rate of decay Δ_2_ could be configured as a valid alternative, thereby enhancing the representativeness of the measurement result. Hence, the current methods neglect the dynamic profile of force application over time, which could provide deeper insights into neuromuscular health, fatigue resistance and recovery. Although testing under dynamic conditions is desirable, this type of measurement is more complex and less straightforward than a static evaluation. The devices that perform this function are more technologically complex, have a higher cost and, above all, require sufficient experience in use and the processing procedure. The main protocols and most of the works in the literature are focused only on the static aspect of this measurement and on the determinations of a single descriptive parameter for HGS. Such dynamics are particularly valuable in clinical settings where they can offer diagnostic and prognostic insights. Moreover, this review has identified a significant gap in the literature concerning the metrological validation of these devices. Few studies ensure measurement traceability to international standards, raising concerns about the accuracy and reliability of the data, especially when such measurements are used to support clinical decisions or to design rehabilitation protocols. This lack of standardization can lead to inconsistencies in data interpretation and affect the comparability of studies, thereby limiting the scientific rigor of research in this field.
(3) Endogenous and exogenous factors of influence—There is also a critical need to define standardized testing protocols, which should be universally applicable, yet adaptable to account for variability among different populations and clinical conditions. Adapting measurement protocols to account for endogenous factors such as age, gender, hand size and body mass, as well as exogenous factors such as job occupation and circadian influences, will improve the accuracy of HGS assessments and may better reflect individual capabilities and limitations, providing a more accurate basis for therapeutic or training interventions. Indeed, all measurements, involving living creatures and specifically humans, require a sufficient degree of individuality to be considered valid. Moreover, the procedure of measurement could better reflect individual capabilities and limitations, thereby providing a more accurate basis for therapeutic or training interventions. Specifically, the sport area is characterized by a significant degree of individuality for its athletes, whereas this orientation tends to be limited in the medical field. Thus, the introduction of some corrective parameters, acting on the attributable HGS, could be desirable. In this regard, it could be suggested that first patients be classified as healthy or pathological. Subsequently, a corrective scale could be implemented in accordance with the physiological characteristics and, if applicable, the specific disease of the subject. Certainly, such an effort should require the collaborative efforts of several professionals and the processing of a considerable amount of data.

The reported limitations underscore the significant research effort still needed in this field. However, the interest in HGS assessment is growing and has accelerated in recent years. This trend suggests that HGS measurements remain an active area of debate and require critical attention, given the importance of the same HGS in supporting medical diagnosis and prognosis.

## Figures and Tables

**Table 1:** Main metrological features of the most common classical dynamometers.

Name, Model	Manufacturer, Country	Measurement Range	Accuracy	Resolution	Size	Weight	References
Jamar, 5030J1	Patterson Medical	0 to 90 kg	±1%	1 kg	102 mm × 216 mm × 318 mm	0.4 kg	[72,77]
Jamar, Smart	Patterson Medical	0 to 90.0 kg	±1%	0.1 kg	102 mm × 267 mm × 356 mm	1 kg	[11,78]
Jamar, Plus +	Patterson Medical	0 to 90.0 kg	±1%	0.1 kg	102 mm × 267 mm × 356 mm	1 kg	[28,79]
T.K.K., 5102	Takei Scientific Instruments (Niigata, Japan)	0 to 100.0 kg	±2.0 kg	0.5 kg	59 mm × 154 mm × 235 mm	0.6 kg	[80,81]
T.K.K., 5401	Takei Scientific Instruments	0 to 100.0 kg	±2.0 kg	0.1 kg	62 mm × 154 mm × 235 mm	0.6 kg	[82,83]
MAP, 130K1	Kern & Sohn (Baden-Württemberg, Germany)	0 to 130.0 kg	-	0.1 kg	55 mm × 102 mm × 212 mm	0.4 kg	[84,85]
MAP, 80K1	Kern & Sohn	0 to 80.0 kg	-	0.1 kg	55 mm × 102 mm × 212 mm	0.4 kg	[23,86]
Camry, EH101	Camry Electronic (Zhaoqing, China)	0 to 90.0 kg	±0.5 kg	0.1 kg	30 mm × 125 mm × 195 mm	-	[26,87]
Camry, EH201R	Camry Electronic	0 to 90.0 kg	-	0.1 kg	78 mm × 139 mm × 227 mm	0.2 kg	[88,89]
Baseline, lite 12-0241	Baseline Instruments (White Plains, NY, USA)	0 to 90 kg	±2%	1 kg	30 mm × 152 mm × 254 mm	1.3 kg	[16,90]
Saehan, SH5001	Saehan (Yongin-si, Republic of Korea)	0 to 90 kg	±1%	1 kg	72 mm × 130 mm × 250 mm	0.7 kg	[18,91]
MicroFET, 4	Hoggan Scientific (Salt Lake City, UT, USA)	0 to 90.72 kg	±1%	0.45 kg	102 mm × 305 mm × 356 mm	1.8 kg	[92,93]

## Data Availability

No new data were created or analyzed in this study.
